# Introgression Lines: Valuable Resources for Functional Genomics Research and Breeding in Rice (*Oryza sativa* L.)

**DOI:** 10.3389/fpls.2022.863789

**Published:** 2022-04-26

**Authors:** Bo Zhang, Ling Ma, Bi Wu, Yongzhong Xing, Xianjin Qiu

**Affiliations:** ^1^National Key Laboratory of Crop Genetic Improvement and National Center of Plant Gene Research, Huazhong Agricultural University, Wuhan, China; ^2^College of Agriculture, Yangtze University, Jingzhou, China

**Keywords:** introgression line, QTL detection, genetic interaction, elite allele identification, breeding by design

## Abstract

The narrow base of genetic diversity of modern rice varieties is mainly attributed to the overuse of the common backbone parents that leads to the lack of varied favorable alleles in the process of breeding new varieties. Introgression lines (ILs) developed by a backcross strategy combined with marker-assisted selection (MAS) are powerful prebreeding tools for broadening the genetic base of existing cultivars. They have high power for mapping quantitative trait loci (QTLs) either with major or minor effects, and are used for precisely evaluating the genetic effects of QTLs and detecting the gene-by-gene or gene-by-environment interactions due to their low genetic background noise. ILs developed from multiple donors in a fixed background can be used as an IL platform to identify the best alleles or allele combinations for breeding by design. In the present paper, we reviewed the recent achievements from ILs in rice functional genomics research and breeding, including the genetic dissection of complex traits, identification of elite alleles and background-independent and epistatic QTLs, analysis of genetic interaction, and genetic improvement of single and multiple target traits. We also discussed how to develop ILs for further identification of new elite alleles, and how to utilize IL platforms for rice genetic improvement.

## Introduction

Introgression lines (ILs) are a set of lines constructed by using a combination of continuous backcrossing and selfing to replace chromosome fragments of the recipient parent (RP) with chromosome fragments of the donor parent (DP), which can directly transfer the desired traits of interest from exotic varieties to adapted varieties. IL sets containing overlapping introgression segments covering the entire genome of DP in the RP background are referred to as chromosome segment substitution lines (CSSLs).

The number of backcrosses, number of backcrossed lines in each generation and number of selfings vary in different rice ILs construction programs, depending on the purpose of ILs construction, crossing methodology, genetic distance between parents, compatibility of genotypes, and number or size of target chromosome segment introgressions (Cavanagh et al., 2008; Balakrishnan et al., 2019). The backcrossed progenies can be selected either by phenotyping or by genotyping during the ILs construction, and then are continuously backcrossed with the RP to produce ILs. When screening progenies of backcrosses based on phenotype, if the trait of interest is controlled by a recessive gene, selfing is required before each backcross to identify plants with the recessive allele of interest (Vogel, 2009). With the development of rice functional genomics and molecular markers, marker-assisted selection (MAS) provides a more direct and effective way to obtain the desired rice ILs (Agarwal et al., 2008), and a series of IL/CSSL libraries have been constructed for QTL identification, gene cloning and variety improvement (Zhang et al., 2021c). The rapid development of high-throughput and high-density SNP genotyping technologies has facilitated the precise identification of introgression segments in ILs (Michael, 2014).

ILs are not only powerful prebreeding tools for broadening the genetic base of existing cultivars, but also valuable resources for quantitative trait loci (QTLs) mapping, gene effects evaluation, favorable alleles identification and genetic interactions analysis. The genetic background noise of ILs with a few chromosome-segment substitutions is low, so ILs with significant differences from the recurrent parents can be rapidly identified by evaluating the phenotype of target trait. Although the construction of ILs is time-consuming and labor-intensive, ILs have been widely reported in cereals, oil crops, vegetables, and commercial fiber crops due to their advantages (Balakrishnan et al., 2019). Harlan and Pope (1922) first used the backcrossing to transfer the smooth awn trait to an elite barley cultivar a century ago. Since then, backcross breeding has gradually developed into a widely used crop breeding method, which is often used to enhance crop resistance to disease and insects to ensure high and stable yield. Rice breeding has undergone two important revolutions including semidwarf breeding and heterosis utilization, both of which are based on gene introgression (Rao et al., 2014; Wu et al., 2018).

Cultivated rice, especially Asian rice, mainly consists of two subspecies, namely *indica* and *japonica* (Kovach et al., 2007; Wang et al., 2018). The two subspecies are further classified into 9 subpopulations with substantial genetic divergence and geographical distribution differences, including XI-1A, XI-1B, XI-2, XI-3, GJ-trp, GJ-sbtrp, GJ-tmp, cA, and cB (Wang et al., 2018). Inter- and intra-specific genetic diversity in mapping and breeding populations is fundamental. To date, a large number of rice ILs have been developed with distant hybridizations or intraspecific crosses (Balakrishnan et al., 2019; Zhang, 2021). The characteristics and construction processes of ILs/CSSLs were well described in previous reviews (Cavanagh et al., 2008; Ali et al., 2010; Balakrishnan et al., 2019). In this paper, we discussed the importance of parental selection during ILs construction, reviewed the recent achievements from rice ILs in functional genomics and molecular breeding, and proposed how to develop ILs for further exploring novel alleles and facilitating genetic improvement of rice.

## Selection of Recurrent and Donor Parents for Introgression Lines Construction

**FIGURE 1:**
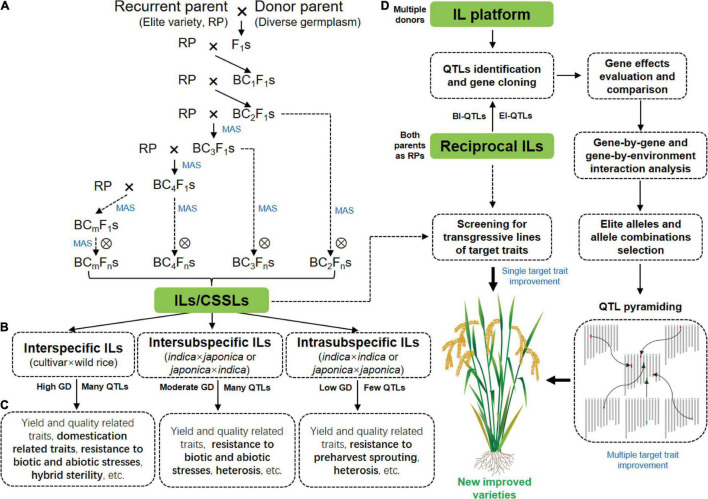
Construction of rice ILs and their application in functional genomics research and breeding. **(A)** A traditional backcross scheme for ILs/CSSLs development with MAS. **(B)** ILs with different crossing types. **(C)** The main traits for genetic dissection by ILs derived from different crosses. The specific traits for the genetic dissection by different ILs are highlighted in bold. **(D)** The strategy of rice breeding by using ILs or IL platform. GD, genetic diversity; BI-QTLs, background-independent QTLs; E-QTLs, epistatic QTLs.

The selection of parents is important for ILs construction because genetic differences between parents are the basis of breeding and functional genomics studies using ILs. A traditional backcross scheme for ILs construction with MAS is showed in Figure 1A. Rice varieties with excellent comprehensive traits but defects in one or few traits are often used as RPs for ILs construction with the goal of breeding improvement, and varieties that excel in these traits are selected as DPs. To date, the excellent *indica* rice varieties, including Huajingxian 74, Zhenshan 97B, 93-11, IR24, and IR64, and the elite *japonica* rice varieties, including Nipponbare, Koshihikari and Taichung 65, have been used as RPs (Table 1). Of course, these varieties are also used as donors in ILs construction. With the reference genomes of parents, ILs/CSSLs are ideal materials with which to comprehensively dissect the genetic basis of agronomic traits, and any genomic variations that cause phenotypic changes can be verified by these materials. Several sets of CSSLs have been constructed using parents with reference genomes (Table 1), such as CSSLs derived from the crosses between Zhenshan 97B and Nipponbare (Li et al., 2011), between Zhenshan 97B and Minghui 63 (Shen and Xing, 2014), and between 93 and 11 and Nipponbare (Zhu et al., 2009; Yuan et al., 2019).

**TABLE 1:** List of introgression lines developed with several elite cultivars as recipient parents.

Recipient parent	Donor parent	Line	Generation	Trait*	References
***Indica***					
Huajingxian 74^#^	*Indica* (cv. IR64, CLSJM) and *japonica* (cv. Suyunuo, IRAT261, Lemont, IAPAR9)	86 SSSLs	BC_3_F_2_, BC_3_F_3_	Heading date, Plant height	He et al., 2005a,b,c
		217 SSSLs	BC_4_F_2_	Tiller and panicle number	Xi et al., 2006; Liu et al., 2008, 2009
	*Indica* (cv. Basmati385)	153 SSSLs	–	Grain width	Wang S. et al., 2012
	*O. meridionalis* (IRGC104093, 105286, 105293, 105291)	99 SSSLs	BC_4_F_2_ to BC_7_F_2_	10 Yield-related traits	He et al., 2017
Zhenshan 97B^#^	*Indica* (cv. Minghui 63^#^)	202 CSSLs	BC_4_F_2_	Heading date, plant height, heterosis	Shen and Xing, 2014; Shen et al., 2014
	*Indica* (cv. Pokkali)	172 ILs	BC_4_F_2_	Plant height	Kovi et al., 2011
	*Japonica* (cv. Nipponbare)	143 CSSLs	BC_4_F_2_	Grain size, seed dormancy, heterosis	Yuan S. et al., 2020; Wang et al., 2021; Xiong et al., 2021
	*O. rufipogon* (IRGC105491)	111 CSSLs	BC_2_F_4_ to BC_6_F_3_	Chlorophyll content	Gao et al., 2020
93-11^#^	*Indica* (cv. PA64s)	81 CSSLs	BC_4_F_2_ to BC_6_F_2_	11 Agronomic traits	Zhang et al., 2019b
		156 CSSLs	BC_3_F_4_ to BC_5_F_3_	8 Yield-related traits, heterosis	Liu et al., 2016; Lin et al., 2020
	*Japonica* (cv. Nipponbare)	103 CSSLs	BC_4_F_2_	Seed shattering, grain size	Zhu et al., 2009
		119 CSSLs	BC_4_F_2_	Tiller angle	Zhao et al., 2012
		122 CSSLs	BC_3_F_2_, BC_4_F_3_	Seed dormancy	Zhang et al., 2020a
		128 CSSLs	BC_4_F_3_ to BC_6_F_2_	Root traits, heterosis	Xu et al., 2010; Tao et al., 2016; Zhou et al., 2016
		55 CSSLs	BC_4_F_2_ to BC_6_F_2_	11 Agronomic traits	Zhang et al., 2019b
	*Japonica* (cv. C418)	108 CSSLs	BC_3_F_8_	Grain weight	Bian et al., 2010
	*O. rufipogon* (DP30)	132 CSSLs	BC_4_F_2_ to BC_6_F_2_	Plant architecture, cold tolerance	Yuan R. et al., 2020
	*O. rufipogon* (CWR276)	198 CSSLs	BC_4_F_5_ to BC_7_F_4_	10 Agronomic traits	Qiao et al., 2016
	*O. rufipogon* (CWR274)	133 CSSLs	BC_3_F_4_ to BC_5_F_3_	Grain size, grain weight	Qi et al., 2017
IR24	*Japonica* (cv. Asominori)	66 CSSLs	BC_1_F_2 (RILs × IR24)_	Ferrous iron toxicity	Wan et al., 2003
		70 CSSLs	BC_2_F_2(RILs × IR24)_	Heading date, grain size	Kubo et al., 2002
	*Oryza minuta* (No. 101133)	131 ILs	BC_4_F_6_	10 Yield-related traits	Guo et al., 2013
IR64	*Japonica* (cv. Koshihikari)	39 CSSLs	–	28 Agronomic traits	Ujiie et al., 2016
	*Japonica* (IRGC23364)	26 CSSLs	BC_4_F_4_	Root angle	Uga et al., 2015
	*Japonica* (cv. Binam)	99 ILs	BC_2_F_8_	Salt tolerance	Zang et al., 2008
	10 Donor varieties	334 ILs	BC_3_F_8_	Spikelet number	Fujita et al., 2012
	*O. rufipogon* (IRGC105491)	105 ILs	BC_2_F_5_	7 Agronomic traits	Cheema et al., 2008
	*O. rufipogon* (W1944, IRGC106148, 105567)	218 CSSLs	BC_3_F_3_ to BC_6_F_3_	–	Singh et al., 2020
	*O. glaberrima* (RAM54, 90)	200 ILs	BC_2_F_4_	Root traits	Kijoji et al., 2014
***Japonica***					
Nipponbare^#^	*Indica* (cv. 93-11)	57 CSSLs	BC_4_F_4_, BC_5_F_3_	Grain weight, panicle architecture, amylose content	Zhang et al., 2011, 2019c; Su et al., 2021
	*Indica* (cv. PA64S)	61 CSSLs	BC_4_F_2_ to BC_6_F_2_	11 Agronomic traits	Zhang et al., 2019b
	*Indica* (cv. Kasalath)	54 CSSLs	–	Root system development	Suralta et al., 2008
	*Japonica* (cv. Koshihikari)	48 CSSLs	BC_4_F_4_	Pre-harvest sprouting	Hori et al., 2010
	*O. rufipogon* (C35)	104 CSSLs	BC_5_F_6_	Panicle-related traits	Ma et al., 2019
Koshihikari	*Indica* (cv. Habataki)	32 CSSLs	BC_3_F_3_ to BC_5_F_4_	Grain quality traits	Murata et al., 2014
	*Indica* (cv. Kasalath)	39 CSSLs	BC_1_F_4_, SBC_3_F_3_	15 Agronomic traits	Ebitani et al., 2005
	*indica* (cv. Takanari)	41 CSSLs	BC_4_F_2_ to BC_4_F_4_	6 Yield-related traits	Takai et al., 2014
	*Indica* (cv. IR64)	43 CSSLs	–	28 Agronomic traits	Ujiie et al., 2016
	*Indica* (cv. Nona Bokra)	44 CSSLs	BC_3_F_3_, BC_3_F_4_	Bacterial seedling rot	Takai et al., 2007; Mizobuchi et al., 2013
		154 CSSLs	BC_3_F_2_	Grain quality traits	Hao et al., 2009
	*Japonica* (cv. Nipponbare)	41 CSSLs	BC_4_F_4_	Pre-harvest sprouting	Hori et al., 2010
	*Japonica* (cv. Owarihatamochi)	44 CSSLs	BC_4_F_5_	Pre-harvest sprouting	Mizuno et al., 2018
	*O. rufipogon* (W0106)	33 CSSLs	BC_4_F_2_ to BC_7_F_2_	10 Agronomic traits	Furuta et al., 2014
	*O. glaberrima* (IRGC104038)	34 CSSLs	BC_4_F_7_ to BC_7_F_7_	10 Yield-related traits	Shim et al., 2010
	*O. barthii* (W0009)	40 CSSLs	BC_4_F_2_ to BC_7_F_2_	10 Agronomic traits	Bessho-Uehara et al., 2017
Taichung 65	*Indica* (cv. ZYQ, DGWG)	10 SSSLs	–	21 Agronomic traits	Liu et al., 2004
	*Indica* (cv. DV85, ARC10313)	89 CSSLs	BC_3_F_5_	–	Yasui et al., 2010
	*O. rufipogon* (W1962)	44 CSSLs	BC_4_F_4_	–	Yamagata et al., 2019
	*O. glaberrima* (IRGC103777)	26 CSSLs	BC_4_F_4_	–	Yamagata et al., 2019
	*O. nivara* (IRGC 105715)	33 CSSLs	BC_4_F_4_	–	Yamagata et al., 2019
	*O. meridionalis* (W1625)	119 CSSLs	BC_4_F_6_	Hybrid breakdown	Munguambe et al., 2021
	*O. longistaminata* (IRGC110404)	40 CSSLs	BC_1_F_7_ to BC_4_F_7_	8 Yield-related traits	Ramos et al., 2016
	*O. longistamina* (W1508)	24 CSSLs	BC_3_F_2_	Anther length	Ogami et al., 2019

*Traits have been evaluated and/or genetically dissected in the corresponding IL populations.

*^#^The reference genomes of these varieties have been released.*

*CLSJM, Chenglongshuijingmi; ZYQ, Zhaiyeqing; DGWG, Dee-Geo-Woo-Gen.*

Wild species of rice contain many useful allelic variations or haplotypes for improving cultivated rice yield and resistance to biotic and abiotic stresses (Kovach et al., 2007). Using wild rice as donors can significantly increase the genetic diversity of cultivars. Although a number of genes from wild species, especially biotic stress resistance genes, have been used in various breeding programs, most of the elite allelic variations in wild rice remains to be exploited (Ali et al., 2010; Li and Zheng, 2013). The genus *Oryza* is composed of 2 domesticated (*O. sativa* and *O. glaberrima*) and 22 wild species (Sanchez et al., 2013). All of six AA-genome wild species and 5 distantly related species with CC, BBCC, CCDD, EE, and FF genomes have been successfully used to construct ILs (Ali et al., 2010; Sanchez et al., 2013; Zhang et al., 2022). To date, more than twenty sets of ILs derived from crosses between wild relatives and cultivated rice have been developed (Ali et al., 2010; Balakrishnan et al., 2019), and some public resource platforms, such as CSSLs created with introgression from four wild rice into the *japonica* rice variety Taichung 65 (Yamagata et al., 2019) and ILs developed from crosses between two varieties (IR64 and Cybonnet) and three wild accessions (Singh et al., 2020), have been initially established.

Although hybrids between *indica* and *japonica* subspecies commonly exhibit varying degrees of hybrid sterility and hybrid breakdown, many researchers have focused on constructing ILs by using *indica-japonica* crosses to perform genetic dissection and improvement of important agronomic traits due to the large degree of genetic divergence between the two subspecies (Balakrishnan et al., 2019). *Japonica* accessions experience harsher high-altitude and/or high-latitude environments, compared with the less harsh but more diverse environments experienced by *indica* rice, resulting in a great difference in agronomic traits between the two subspecies (Wang et al., 2018). Some favorable alleles introgressed between subpopulations, such as the long-grain haplotype of *GS3* (Fan et al., 2006), the wide-grain haplotype of *qSW5/GW5* (Shomura et al., 2008; Weng et al., 2008), the low-amylose haplotype of *Wx* (Tian et al., 2009) and the haplotype of *OsNRT1.1B* with high nutrient use efficiency (Hu et al., 2015), are of great significance for the improvement of rice agronomic traits.

Rice subpopulations originating in specific regions, such as cA and cB, have a larger number of “private” alleles than other subpopulations (Wang et al., 2018). Subpopulation cA encompasses the Aus, Boro and Rayada ecotypes from Bangladesh and India and subpopulation cB comprises the famous Basmati and Sadri aromatic varieties (Wang et al., 2018). The two subpopulations show many elite characteristics, such as the early maturity and drought and heat resistance of Aus varieties and superb fragrance and good cooking quality of aromatic varieties (Civáò et al., 2015). Several Aus varieties, such as Nagina 22 (Lin et al., 2015) and Chuan 7 (Zhang et al., 2021a), have been used as donors to construct ILs, while the aromatic varieties preferred for consumption, such as Khao Dawk Mali 105 (Nounjan et al., 2016; Chutimanukul et al., 2018), Basmati (Grover et al., 2020; Yadav et al., 2020), and Mushk Budji (Khan et al., 2018), are usually used as RPs.

In addition, African cultivated varieties (*Oryza glaberrima*) with an independent origin of domestication are also a valuable exotic gene pool for Asian cultivated rice and have been used as DPs for IL development (Li et al., 2015). The interspecific upland New Rice for Africa (NERICA) varieties have been developed by introgression of chromosome segments from *O. glaberrima* (CG 14) into three *japonica* rice varieties. Blending traits from the African traditional and Asian varieties, NERICA varieties have superior agronomic characteristics such as better weed resistance, resilience against major African biotic and abiotic stresses, high fertilizer returns, and high yields (Jones et al., 1997; Kijima et al., 2006; Fukuta et al., 2012).

ILs constructed by using interspecific and intersubspecific crosses can significantly expand the gene pools of RPs and the large genetic differences between parents also increase the available molecular markers for MAS, which is conducive to mining QTLs. However, the cross incompatibility and F_1_ hybrid sterility increase the difficulties of interspecific and intersubspecific ILs/CSSLs construction (Ali et al., 2010). In contrast, ILs from intrasubspecific crosses are easier to construct but have lower genetic diversity. A number of intrasubspecific ILs have been developed by using elite *indica* or *japonica* varieties as the RPs (Table 1).

## Progress in Using Introgression Lines in Rice Functional Genomics Research

ILs are powerful tools with which to dissect complex traits into a set of monogenic loci, and are appropriate for detecting QTLs with both large and small effects (Xi et al., 2006). Over the past three decades, a large number of IL sets derived-from different crosses have been constructed for mining QTLs/genes for agronomic traits (Figures 1B,C and Table 1).

### Genetic Dissection of Complex Traits by Introgression Lines With the Wild Relatives as Donor Parents

Wild species have acquired many elite alleles for resistance to biotic and abiotic stresses during the long period of natural selection (Atwell et al., 2014; Mammadov et al., 2018). A large number of IL libraries constructed from wild and cultivated rice were used to detect QTLs for cold (Yuan R. et al., 2020), heat (Lei et al., 2013; Prasanth et al., 2016; Cao et al., 2020), drought (Zhang et al., 2006; Zhou et al., 2006), and salt (Tian et al., 2011; Yang et al., 2012) tolerance. For example, a set of 132 CSSLs were developed from a cultivated rice, 93–11, and the Guangxi common wild rice (GXCWR), and the cold-tolerance QTL *qCT2.1* on chromosome 2 was identified using the secondary mapping population (Yuan R. et al., 2020). Using a set of 90 ILs derived from the cross between Teqing and Yuanjiang common wild rice (YJCWR), five QTLs related to heat response (*qHST1-1*, *qHST1-2*, *qHST2*, *qHST3*, and *qHST8*) in the seedling stage were detected, and *O. rufipogon*-derived alleles at one locus reduced sensitivity to heat (Lei et al., 2013). QTLs for resistance to biotic stress, such as that imposed by the green rice leafhopper (Thein et al., 2019), rice stripe necrosis virus (Gutiérrez et al., 2010) and rice blast fungus (Xu et al., 2015), were also mapped using ILs carrying wild rice donor fragments in cultivated rice backgrounds.

ILs developed from wild and cultivated rice are valuable materials for the genetic dissection of domestication-related traits such as an erect growth habit, inflorescence architecture, and awn length. Several domestication genes have been cloned using such IL-derived populations (Table 2). *PROG1*, a prostrate growth gene, was identified in a set of ILs developed from Teqing and YJCWR. Its non-functional allele determines the critical transition of rice from prostrate to erect growth (Tan et al., 2007, 2008). Using the same IL sets, a domestication gene, *OsLG1*, controlling inflorescence architecture was cloned (Zhu et al., 2013). Using a set of 354 ILs derived from the cross between 93 and 11 and YJCWR, an IL with long and barbed awns was identified. Further fine-mapping indicated that a frameshift deletion in *LABA1* of cultivated rice reduces the cytokinin concentration in awn primordia, disrupting barb formation and awn elongation (Fu et al., 2010; Hua et al., 2015). Another domestication gene controlling plant architecture, *TIG1*, was also cloned in an IL population with the wild rice (W2014) segments in the 93–11 background (Zhang et al., 2019d).

**TABLE 2:** List of genes with natural variations identified by ILs and cloned by IL-derived populations in rice.

Gene	Locus	Recipient parent	Donor parent	Trait	References
*NOG1*	LOC_Os01g54860	*Indica* (cv. Guichao 2)	*O. rufipogon* (DXCWR)	Grain number	Huo et al., 2017
*OsGUX1*	LOC_Os01g65780	*Indica* (cv. Zhenshan 97)	*O. rufipogon* (ACC10)	Chlorophyll content	Gao et al., 2020
*OsHMA4*	LOC_Os02g10290	*Japonica* (cv. Lemont)	*Indica* (cv. Teqing)	Copper accumulation	Huang et al., 2016
*CAL1*	LOC_Os02g41904	*Japonica* (cv. Chunjiang06)	*Indica* (cv. Tainan1)	Cadmium accumulation	Luo et al., 2018
*DTH3*	LOC_Os03g03070	*Indica* (cv. Huajingxian74)	Multiple varieties	Heading date	Zhu et al., 2018
*BOC1*	LOC_Os03g12820	*Indica* (cv. Teqing)	*O. rufipogon* (YJWCR)	Callus browning	Zhang et al., 2020b
*TT1*	LOC_Os03g26970	*Japonica* (cv. Wuyunjing)	*O. glaberrima* (CG14)	Thermotolerance	Li et al., 2015
*GNP1*	LOC_Os03g63970	*Japonica* (cv. Lemont)	*indica* (cv. Teqing)	Grain number	Wu et al., 2016
*CTB4a*	LOC_Os04g04330	*Japonica* (cv. Towada)	*japonica* (cv. KMXBG)	Cold tolerance	Zhang et al., 2017
*An-1*	LOC_Os04g28280	*Indica* (cv. Guangluai4)	*O. rufipogon* (W1943)	Awn length; Grain size	Luo et al., 2013
*LABA1/An-2*	LOC_Os04g43840	*Indica* (cv. 93-11)	*O. rufipogon* (YJWCR)	Awn length	Hua et al., 2015
*GW5*	LOC_Os05g09520	*Japonica* (cv. Asominori)	*indica* (cv. IR24)	Grain width	Weng et al., 2008
*OsEBS*	LOC_Os05g51360	*Indica* (cv. Guichao2)	*O. rufipogon* (DXCWR)	Plant biomass; Spikelet number	Dong et al., 2013
*Hd17*	LOC_Os06g05060	*Japonica* (cv. Nipponbare)	*Japonica* (cv. Koshihikari)	Heading date	Matsubara et al., 2012
*GW6*	LOC_Os06g15620	*Indica* (cv. Huajingxian74)	*Japonica* (cv. Nanyangzhan)	Grain width	Shi et al., 2020
*PROG1*	LOC_Os07g05900	*Indica* (cv. Teqing)	*O. rufipogon* (YJWCR)	Prostrate growth	Tan et al., 2008
*OsHMA3*	LOC_Os07g12900	*Indica* (cv. Huajingxian74)	*Indica* (cv. BG367)	Cadmium accumulation	Sui et al., 2019
*COS1/FZP*	LOC_Os07g47330	*Japonica* (cv. C418)	*O. rufipogon* (DXCWR)	Inflorescence branching	Huang et al., 2018
*Hd18*	LOC_Os08g04780	*Japonica* (cv. Koshihikari)	*japonica* (cv. Hayamasari)	Heading date	Shibaya et al., 2016
*TIG1*	LOC_Os08g33530	*Indica* (cv. 93-11)	*O. rufipogon* (W2014)	Tiller angle	Zhang et al., 2019d
*GAD1*	LOC_Os08g37890	*Indica* (cv. NA93-11)	*O. rufipogon* (W2014)	Awn length; Grain size	Jin et al., 2016
*OsSPL16/GW8*	LOC_Os08g41940	*Indica* (cv. Huajingxian74)	*indica* (Basmati385)	Grain width	Wang S. et al., 2012
*GS9*	LOC_Os09g27590	*Japonica* (cv. Nipponbare)	Qingluzan11	Grain size	Zhao et al., 2018
*DTE9/OsMADS8*	LOC_Os09g32948	*Japonica* (cv. Hwayeong)	*O. rufipogon* (W1944)	Hybrid weakness	Kim et al., 2021
*TAC1*	LOC_Os09g35980	*Indica* (cv. IR24)	*japonica* (cv. Asominori)	Tiller angle	Yu et al., 2007
*OsGluA2*	LOC_Os10g26060	*Japonica* (cv. Sasanishiki)	*Indica* (cv. Habataki)	Grain protein content	Yang et al., 2019
*OsLTPL159*	LOC_Os10g36160	*Indica* (cv. Guichao2)	*O. rufipogon* (DXCWR)	Cold tolerance	Zhao et al., 2020
*GL10/OsMADS56*	LOC_Os10g39130	*Indica* (cv. Huajingxian74)	*Japonica* (cv. Lemont)	Grain length	Zhan et al., 2022
*NRT1.1B*	LOC_Os10g40600	*Japonica* (cv. Nipponbare)	*Indica* (cv. IR24)	Nitrogen-use efficiency	Hu et al., 2015
*OsGH3.13*	LOC_Os11g32520	*Indica* (cv. Zhenshan 97)	*Japonica* (cv. Nipponbare)	Grain length	Wang et al., 2021
*TOND1*	LOC_Os12g43440	*Indica* (cv. Teqing)	*O. rufipogon* (YJWCR)	Tolerance to nitrogen deficiency	Zhang et al., 2015

*DXCWR, Dongxiang common wild rice; YJWCR, Yuanjiang common wild rice; KMXBG, Kunmingxiaobaigu.*

There has also been a burst of studies on mapping novel genomic regions and QTLs for yield-related traits (Furuta et al., 2014, 2016; Ma et al., 2016; Qiao et al., 2016; Rao et al., 2018) and quality traits (Garcia-Oliveira et al., 2009; Fasahat et al., 2012; Yun et al., 2016; Qi et al., 2017) by using IL populations derived from the crosses between wild and cultivated rice, generally with the elite varieties as RPs. For example, using 33 CSSLs of *O. rufipogon* (W0106) in the background of the elite *japonica* rice variety Koshihikari, a total of 15 major QTLs for eight yield-related traits were detected, and a novel QTL controlling the number of grains per panicle were identified on chromosome 10 (Furuta et al., 2014). In the same background of Koshihikari, a set of 26 CSSLs with segments from *O. nivara* were constructed, and new QTLs associated with yield-related traits were identified (Furuta et al., 2016). A set of 198 CSSLs were developed by introgressing *O. rufipogon* segments into the background of the elite *indica* rice variety 93–11, and a new QTL associated with the heading date was detected in a 78-kb region on chromosome 10 (Qiao et al., 2016). Similarly, a set of 131 ILs were developed by introducing *O. nivara* segments into the 93–11 background, and 65 QTLs for 13 yield-related traits were detected by bin-map, with ∼36.9% of the alleles at the detected QTLs from *O. nivara* leading to improved yield-associated traits (Ma et al., 2016). QTLs associated with 12 grain quality traits were detected using 96 ILs developed from an interspecific cross between the Korean elite *japonica* rice variety Hwaseong and *O. rufipogon* (IRGC 105491). A total of 48 QTLs for these traits were identified, and most wild alleles of these detected QTLs had negative effects on the traits (Yun et al., 2016).

In addition to the traits mentioned above, IL sets developed from wild and cultivated rice have also been used for genetic dissection of other traits, such as interspecific hybrid sterility (Yang et al., 2016; Li et al., 2018a; Zhang et al., 2018), anther length (Ogami et al., 2019), the stigma exsertion rate (Tan et al., 2020), out-crossing rate (Prahalada et al., 2021), and photosynthetic efficiency (Rao et al., 2018).

### Genetic Dissection of Complex Traits by Using Introgression Lines Derived From Intersubspecific Crosses

Multiple IL sets derived from *indica-japonica* crosses have been developed to mine QTLs for rice yield-related traits (Ando et al., 2008; Sun et al., 2017; Mitsuya et al., 2019), quality-related traits (Hao et al., 2009; Sun et al., 2015; Wang et al., 2021), and resistance to biotic (Park et al., 2007) and abiotic (Zang et al., 2008; Zhao et al., 2016; Feng et al., 2018) stresses. Several QTLs for these traits have been detected and causal natural variations for the detected QTLs have been identified by using IL-derived F_2_ populations (Table 2). For example, two sets of reciprocal ILs derived from a *japonica* rice variety Lemont and an *indica* variety Teqing were constructed, and multiple QTLs for grain number per panicle (GNP) were identified form these two ILs. Among these detected QTLs, a stable QTL *GNP1* was further finely mapped to a 33.7-kb region and a GA biosynthesis gene *GA20-oxidase 1* was identified as the candidate gene for *GNP1* (Wu et al., 2016). QTL analysis of seven panicle- and grain-related traits was performed in a set of genotype-defined CSSLs derived from a cross of two genome-sequenced varieties, Nipponbare and Zhenshan 97, and a total of 43 QTLs for these traits were identified. Furthermore, the novel locus *qGL11* for grain length and thousand-grain weight was finely mapped to a 25-kb region, and the IAA-amido synthetase gene *OsGH3.13* was verified as the causal gene for *qGL11* (Wang et al., 2021).

Heterosis is one of the most important characteristics of F_1_ plants derived from *indica-japonica* crosses (Dan et al., 2014). Many ILs and IL-derived backcross and/or testcross populations have been used to dissect the genetic basis of heterosis (Xin et al., 2011; Wang Z. et al., 2012; Tao et al., 2016; Xiong et al., 2021; Yang et al., 2021c). For example, a set of 70 ILs and corresponding testcross F_1_ populations were developed from a cross between the *indica* rice variety IR24 (RP) and *japonica* rice variety Asominori to investigate heterotic loci (HLs) associated with six yield-related traits (Xin et al., 2011). A total of 41 HLs were detected on the basis of mid-parent heterosis values with single-point analysis, and most of QTLs were overdominant, suggesting that heterotic effects at the single-locus level are mainly overdominant in rice (Xin et al., 2011). Tao et al. (2016) constructed a set of 128 CSSLs derived from a cross between the *indica* rice variety 93–11 and *japonica* rice variety Nipponbare to investigate the genetic basis of heterosis. Using the testcross populations derived from CSSLs and three P-TGMS lines, a total of 97 HLs associated with yield components were detected. Genetic analysis revealed that the contribution of different genetic effects to heterosis might vary among traits (Tao et al., 2016; Yang et al., 2021c). In a recent study, *Ghd8* was identified and verified as a major HL for yield components by using a set of intersubspecific CSSLs and two test populations (Xiong et al., 2021). This HL has also been identified in the two-line rice hybrid system (Li et al., 2016), indicating its important role in hybrid breeding.

QTLs have been mapped in ILs derived from *indica-japonica* crosses for other traits, including hybrid sterility (Zhao et al., 2011), nutrient utilization (Kabange et al., 2021), seed germination and dormancy (Li et al., 2011; Zhang et al., 2020a), mature seed culturability (Zhao et al., 2009), carbon isotope discrimination (Takai et al., 2009), lodging resistance (Mulsanti et al., 2018), and cell wall characteristics (Xu et al., 2017).

### Genetic Dissection of Complex Traits by Using Introgression Lines Derived From Intrasubspecific Crosses

ILs developed from intrasubspecific crosses are favored by breeders because there are no incompatibility barriers in these crosses. Although the genetic diversity within rice subspecies is lower than that between subspecies, there are still large phenotypic differences among varieties from different regions with different genealogical relationships in the same subspecies. Several IL libraries from intrasubspecific crosses have been developed in rice to genetically dissect complex traits such as yield-related traits (Shen and Xing, 2014; Hori et al., 2021a; Kato and Hirayama, 2021), quality-related traits (Yang et al., 2021b), root traits (Anis et al., 2019), resistance to preharvest sprouting (Hori et al., 2010; Mizuno et al., 2018), and heterosis (Lin et al., 2020). For example, a set of 202 CSSLs of an elite hybrid, Shanyou 63, were developed in the Zhenshan 97 background, and QTLs for heading date and plant height were detected (Shen and Xing, 2014; Shen et al., 2014). A total of 15 partial dominance QTLs for plant height were identified in 15 CSSL-derived F_2_ populations, and these QTLs were further identified as HLs for plant height that acted dominantly and epistatically (Shen et al., 2014). Furthermore, multiple QTLs and HLs for yield and spikelets per panicle were identified in these CSSLs, and the hybrids pyramiding these detected HLs in the combined parental genome background showed yield performance similar to that of Shanyou 63, indicating that heterosis might be successfully achieved by manipulating several major dominant HLs (Shen et al., 2022).

### Identification of Elite Alleles by Ranking the Effects of Different Alleles With Introgression Lines

ILs derived from advanced backcrosses generally carry only one or a few chromosome fragments from the donor, which greatly minimizes the genetic background noise and facilitates the evaluations of the genetic effects of QTLs/genes on corresponding traits. The target introgressed segment of an IL is homozygous, conferring its genomic stability, so ILs can be repeatedly planted in multiple sites to evaluate the genetic effects of QTLs across different environments (Zhou et al., 2017). A set of ILs covering the entire DP genome can be applied to identify multiple QTLs associated with the trait of interest, and the genetic effects of these detected QTLs can be compared based on their additive effect in the same background, especially using single segment substitution lines (SSSLs) (Ye et al., 2010; Zhu et al., 2018). ILs are not only beneficial for comparing the genetic effects of different QTLs in the same background but also beneficial for comparing the effects of different alleles at one QTL in the fixed background, so as to identify the favorable alleles or allele combinations. For example, the heading date QTL *qHD-3* was identified by using a library consisting of 1123 CSSLs from multiple donors in the same genetic background, Huajingxian 74 (HJX74). Compared with the HJX74 allele, the *qHD-3* allele from donor variety Lemont delayed rice heading, while *qHD-3* from another donor parent, IR64, promoted rice heading. Further sequencing analysis revealed that different variants of *DTH3* were identified between Lemont and IR64 (Zhu et al., 2018). To investigate the interaction effect of *Ghd8* on yield mid-parent heterosis (MPH), Xiong et al. (2021) constructed five near-isogenic lines (NILs) with each carrying an introgression segment covering *Ghd8* from a particular donor in the same background of ZS97, and identified the combination (*Ghd8*^ACC10^*/Ghd8*^MH63^) with the highest MPH for both spikelet number and grain yield per plant using a half-diallel mating design.

### Identification of Background-Independent and Epistatic Quantitative Trait Loci Using Reciprocal Introgression Lines

Reciprocal ILs developed in both parental backgrounds have the advantage of enabling evaluation of differences in allelic effects of QTLs in both genetic backgrounds (Kubo et al., 2002). Reciprocal ILs are not only conducive to the detection and fine mapping of QTLs that have both large and small effects but also appropriate for evaluating gene activity as a single factor or in epistatic interactions (Takai et al., 2014). Reciprocal ILs have been used to identify background-independent QTLs (BI-QTLs) and epistatic QTLs (E-QTLs) for grain yield- and quality-related traits (Takai et al., 2014; Qiu et al., 2017; Hori et al., 2021b), salt tolerance (Cheng et al., 2012), lodging resistance (Ookawa et al., 2016), the leaf net photosynthetic rate (Adachi et al., 2019), and sink- and source-related traits (Wang et al., 2020). For example, using the reciprocal CSSLs derived from a cross between MH63 and 02428, a total of nine BI-QTLs for appearance quality were identified. Thirteen and 10 stably expressed QTLs (SE-QTLs) were also detected in the MH63 and 02428 backgrounds, respectively (Qiu et al., 2017). Compared with E-QTLs, BI-QTLs, and SE-QTLs are more easily applied for rice genetic improvement because their function does not depend on background or the environment.

### Genetic Interaction Analysis Conducted by Using Introgression Lines and Introgression Line-Derived Populations

Most agronomic traits of crops are complex traits controlled by multiple genes and affected by environmental factors. Understanding the genetic and environmental bases of QTL × QTL and gene-by-environment (G × E) interactions is of fundamental importance in plant breeding (Monteverde et al., 2019). As in recombinant inbred lines (RILs), genetic interactions between QTLs/genes, such as additive-by-dominance and dominance-by-dominance interactions, also cannot be analyzed using ILs because all sites in ILs are homozygous. However, the offspring-separated populations derived from the crosses between ILs or backcrosses between ILs and RPs are ideal populations for genetic interaction analysis due to their low genetic background noise. Genetic interaction analysis among four major rice heading date genes, *Ghd7*, *Ghd8*, *OsPRR37*, and *Hd1*, was performed in a 4-gene segregating population in the near-isogenic background under both natural long-day (NLD) and natural short-day (NSD) conditions (Zhang et al., 2019a). Tetragenic, trigenic and digenic interactions among these four genes for heading date were observed under both conditions but were more significant under NLD conditions. Further analysis showed that the differences in digenic interactions between *Ghd7* and *OsPRR37* under different conditions were essential to the alternative function of *OsPRR37* (Zhang et al., 2019a). Genetic interaction analysis between QTLs for other agronomic traits, such as plant height (Shen et al., 2014), grain size (Xia et al., 2018), tillering (Zhou et al., 2020), cold tolerance (Liang et al., 2018), and drought tolerance (Yadav et al., 2019), was also performed in IL-derived populations.

Most the traits with low heritability are easily affected by the environment, and there is an obvious interaction between gene and the environment. Understanding G × E interactions can help breeders in deciding which QTL to use in their breeding programs while tailoring crop cultivars for specific or more diverse environments (Liu et al., 2008). G × E interactions were analyzed in ILs or IL-derived populations planted in a variety of environments. To dissect the genetic basis of the genetic main effect (G) and G × E interaction effect (GE) for panicle number (PN) in rice, a population consisting of 35 SSSLs derived from originating from crosses between the RP, HJX74, and 17 DPs was grown in six cropping season environments (Liu et al., 2008). The total genetic effect was partitioned into G and GE by using the mixed linear-model approach, and then QTL analyses of these effects were conducted separately. A single QTL effect was divided into two components: The additive effect (**a**) and additive × environment interaction effect (**ae**). A total of 18 QTLs for PN were identified, and three types of QTLs were suggested according to their effects expressed. Two QTLs expressed stably across environments due to the association with only **a**, nine QTLs with only **ae** were unstable, and the remaining seven QTLs were identified with both **a** and **ae**, which were also unstable across environments (Liu et al., 2008). Notably, when QTLs have large **ae** values in opposing directions in different environments, selection for an allele with favorable effects in certain environments could lead to undesired results in other environments.

## Introgression Lines Are Valuable Resources for Rice Breeding

Rice breeding is based on the use of valuable alleles and elite germplasm resources. Fortunately, rice has a large number of germplasms containing all kinds of excellent genes for high yield, good quality, resistance to biotic and abiotic stresses, and etc. (Tanksley and McCouch, 1997). Transferring these excellent genes into modern varieties absolutely could broaden the gene pool and greatly improve the productivity. However, many of these valuable alleles have linkage drags (Ali et al., 2010). IL populations have the advantage of breaking the linkage between valuable genes and linkage drags. To date, many traits in rice have been greatly improved by developing ILs in the backgrounds of modern varieties (Hu et al., 2016; Balakrishnan et al., 2019; Zhang, 2021; Zhang et al., 2021c).

### Single Target Trait Improvement by Using Introgression Lines

Each line of ILs carries only one or a few donor chromosome segments, which usually affects only one or a few traits of the RP. Therefore, using ILs to improve single-target trait in rice is efficient. In the elite genetic backgrounds, IL with improved agronomic performance can be released as a new variety.

Increasing rice yield to meet the need for a rapidly growing global population is one of the most important goals of rice breeding. Many yield-related QTLs/genes have been introgressed into elite backgrounds to increase rice yield. For example, *GN4-1*, a QTL for grain number per panicle, was introgressed into ZH8006 from WYJ6, and grain yield increased by more than 10% (Zhou et al., 2018). To achieve the high yield potential in Kongyu 131, a minute chromosome fragment carrying the favorable *Gn1a* allele from the donor parent GKBR was introgressed into the genome of Kongyu 131, which resulted in larger panicles and a subsequent yield increase in the new Kongyu 131 (Feng et al., 2017). In addition, introgression of a functional epigenetic *OsSPL14*^WFP^ allele into elite *indica* cultivars can greatly improve panicle traits and grain yield (Kim et al., 2018).

With the increasing development of people’s living standards, rice quality has received attention from both rice producers and consumers. There have been many successful cases of improving rice grain quality through ILs. For example, to improve the eating and cooking quality of Zhenshan 97 and its hybrid Shanyou 63, the *Wx/Waxy* gene region from Minghui 63, a restorer line with medium amylose content (AC), soft gel consistency (GC), and high gelatinization temperature (GT), was introgressed into Zhenshan 97B and then transferred into Zhenshan 97A (Zhou et al., 2003). In recent years, the discovery of different alleles of *Wx* and *ALK* has provided more choices for rice grain quality improvement via gene introgression (Chen et al., 2020; Huang et al., 2021; Zhang et al., 2021b; Zhou et al., 2021). *GS9* is an important gene for both grain shape and chalkiness, and appearance quality could be largely improved by developing an IL for this gene (Zhao et al., 2018, 2021).

Biotic and abiotic stresses are large threats to rice production, leading to serious yield losses every year. ILs constructed by introgressing novel genes into modern varieties are an effective tool for solving this problem. For example, by combining a backcross breeding strategy combined with MAS, a total of 12 blast resistance genes were introgressed into one maintainer line of cytoplasmic male sterility (CMS) and three photo-thermo genetic male sterility (P-TGMS) lines. The blast resistance of these lines was significantly improved, while other traits were not changed (Jiang et al., 2012, 2015, 2019). Using the same strategy, brown planthopper resistance has been improved by introgressing *Bph3*, *Bph14*, *Bph15*, *Bph18*, *Bph20*, *Bph21*, and *Bph33* into 93–11 and Jin23B (Hu et al., 2013, 2018; Jiang et al., 2018), and bacterial blight resistance has been improved by pyramiding *Xa4*, *xa5*, *xa13*, and *Xa21* (Huang et al., 1997). In the two IL populations developed with C258 and ZGX1 as RPs and IR75861 as the DP, 12 and 8 lines had higher salt tolerance than the RPs, respectively, and they could be directly planted into environments characterized by salt stress (Qiu et al., 2015).

The excess application of fertilizers in rice production not only increases cost, but also causes severe environmental problems (Zhang et al., 2020c). Breeding rice varieties with high nitrogen-use efficiency (NUE) is of great significance to the practice of Green Super Rice (Yu et al., 2020). GRF4 promotes and integrates nitrogen assimilation, carbon fixation, and growth, whereas DELLA inhibits these processes. Introgression of *GRF4* into the elite variety 93–11 carrying *sd1* can significantly improve its NUE, thereby increasing yield (Li et al., 2018b). *NAL1* and the gain-of-function mutation *dep1-1* have been reported to play important roles in N-responsive tillering regulation, and pyramiding of the *dep1-1* and *NAL1*^NJ6^ alleles can achieve sustainable improvements in NUE and grain yield in *japonica* rice breeding (Sun et al., 2014; Xu et al., 2019). The introgression of favorable alleles of other NUE-related genes such as *OsTCP19* and *OsSBM1* also showed great potential for genetic improvement of high NUE in rice (Liu et al., 2021; Xu et al., 2021).

### Multiple Target Trait Improvement by Using Introgression Lines

To meet people’s diverse demands for rice, modern rice varieties should be improved mainly by multiple locus introgression to improve the overall features of rice in terms of grain yield and quality, resistance to biotic and abiotic stresses and nitrogen use efficiency, etc. For example, Luan et al. (2019) used four SSSLs with HJX74 as the RP to develop a new maintainer named H131B with a suitable heading date, good appearance and eating and cooking quality, and fragrant smell by pyramiding *MADS50*, *gs3*, *fgr*, *Wx*^gl^, and *ALK*. Dixit et al. (2020) developed seven ILs possessing a combination of seven to ten QTLs for resistance to different biotic and abiotic stresses by using the MAS breeding method in the background of Swarna. These ILs were superior to the respective RPs in terms of agronomic performance and possessed superior grain quality. Luo et al. (2016) successfully developed a new restorer line, WH6725, with disease resistance to rice blast and bacterial blight, tolerance to submergence and an aromatic fragrance by pyramiding *Xa27*, *Pi9*, *Sub1A*, and *badh2.1*. Through genotyping-based identification and backcrossing with MAS, Zeng et al. (2017) introgressed 21 favorable alleles from NIP and 9311 into Teqing and then developed three lines that exhibited higher yield potential and better grain quality than their parental varieties and the super-hybrid rice Liang-you-pei-jiu. Guangzhan 63-4S is an elite two-line thermos-sensitive genic male sterile line. However, it is highly susceptible to blast and bacterial blight. Two BC_2_ ILs were developed to introgress *Xa7* for bacterial blight and *Pi-2* for blast resistance. Then, the ILs were crossed and pyramided with these two genes. A new two-line thermosensitive genic male-sterile line named Hua1228S that was resistant against both rice blast and bacterial blight was developed (Mi et al., 2018).

### Rice Breeding Improvement by Using Introgression Line Platform

IL platform developed by multiple donors in an elite genetic background facilitates the discovery of new QTLs and identification of multiple alleles of a gene. IL platform can be used to identify the optimal allele or allele combination by evaluating their genetic effects in the fixed background and then improve varieties by QTL pyramiding (Zhang, 2021; Figure 1D). In addition, new varieties can be directly developed by screening transgressive lines of target traits in the IL platform (Figure 1D). HJX74 is an elite variety from South China. A single segment IL platform including more than 2000 ILs was developed with HJX74 as the RP and more than 40 varieties as DPs (Luan et al., 2019; Zhang, 2021). A large number of important genes were identified using this platform (Wang S. et al., 2012; Wang et al., 2015; Yang et al., 2021a). In addition, a series of varieties have been bred by pyramiding different favorable genes in this platform, such as three-line maintainer lines (H121B, H131B, HZB, HBB, E5-HXB, and E5-HBB) (Luan et al., 2019), black rice (Huaxiaohei1 and Huaxiaohei 2), red rice (Huaxiaohong1 and Huaxiaohong 2), and high-quality rice (Huabiao1 and Huabiao 3) (Zhang, 2021). Huanghuazhan (HHZ) is an elite rice variety widely planted in the middle and downstream regions of the Yangtze River and in South China. An IL platform including 496 ILs were developed using HHZ as the RP and 8 varieties as DPs (Ali et al., 2017). Most ILs showed significantly higher yields than the parents under both abiotic stress conditions (salt and drought) and non-abiotic stress conditions (submergence). Ultimately, at least six ILs were directly released as new green super rice varieties in the Philippines and Pakistan and have been grown on more than 1 million ha of land in different ecosystems (Ali et al., 2017).

## Conclusion and Perspectives

ILs simplify the genetic dissection of complex traits and accelerate whole-genome large-scale gene discovery in rice. To date, a large number of the natural variations associated with agronomic traits in rice have been identified from ILs and then cloned using IL-derived mapping populations (Li et al., 2018c; Zhang, 2021; Table 2). The primary mapping populations may be more cost-effective for large-effect QTLs mapping, but ILs have a greater advantage in detecting minor QTLs because of their low genetic background noise. The resolution of QTL mapping using ILs is low due to the large size of the introgression segments and the selective abandonment of some recombination events (lack of lines) during the construction of ILs (Cavanagh et al., 2008; Balakrishnan et al., 2019). Substitution mapping of QTLs using multiple ILs is an effective way to improve the mapping resolution (Zhang, 2021). In addition, any two ILs carrying different QTLs/genes controlling the same trait can be used for the epistatic interaction detection with their offspring-segregated population, which can provide useful information on how to select favorable alleles and allele combinations in rice molecular design breeding.

A large number of genetic dissections of traditional agronomic traits have been performed in rice (Li et al., 2018c; Wei et al., 2021), so it is difficult to further mine new QTLs/genes for these traits. While some rice subpopulations or distant relatives, such as cA, cB, and Africa cultivated rice, with narrow geographic or relatively independent origins, may carry many private (rare) genes and novel natural variations due to the differences in domestication and environmental pressures (Li et al., 2015; Bai et al., 2017; Wang et al., 2018). Accessions from these subpopulations were rarely used in the past, but are encouraged used as donors for constructing ILs in the future because more new QTLs might be detected using this kind of ILs. Many ILs have been constructed using AA-genome wild species as donors, but there are few successful cases of constructing ILs using other wild *Oryza* species as donors due to the strong incompatibility barriers (Sanchez et al., 2013). These wild species not only have strong biotic and abiotic stress resistance, but also have unique and excellent traits, such as the early morning flowering trait in CC-genome wild rice *Oryza officinalis* and the high biomass production in CCDD-genome wild species (Sanchez et al., 2013; Hirabayashi et al., 2015). Thus, using more different wild rice species as genetic resources to broaden the gene pool of cultivars by constructing ILs is of great significance, but it is also full of challenges. Moreover, multiple donors from different wild/cultivated rice species and subpopulations are also encouraged to develop IL platforms in one genetic background for new alleles detection. To date, the third-generation sequencing technology makes the cost of genome assembly dramatically decreased, a large number of rice varieties have been deeply sequenced, and reference genomes of dozens of varieties (including wild and cultivated rice) have been assembled (Wang et al., 2018; Wing et al., 2018; Qin et al., 2021). Thus, the accessions used for ILs development are suggested to generate the genome reference to facilitate the discovery of genetic variations between the recurrent and donor parents. Based on the difference of gene annotations between parents, the ILs contained the target genes can be used to precisely examine the potential target traits and rapidly verify the valuable natural variations. In addition, ILs combined with high-throughput phenotyping platform are encouraged to detect QTLs for traits measured dynamically and non-destructively, especially for the root associated traits and abiotic stress tolerance, which are hard to precisely measure by traditional ways (Yang et al., 2014).

As important prebreeding materials, ILs facilitate the genetic improvement of rice via gene transfer and pyramiding. However, QTL pyramiding for rice variety improvement requires multiple crosses and the avoidance of genetic drag, which usually requires several generations and large populations (Zhang et al., 2021c). Applying the IL platform in an elite background can accurately reveal multiple favorable alleles that can be used to improve the RP. This platform can also be used to evaluate the genetic effects of different alleles at the same locus and the effects of different allele combinations to identify the optimal allele or combination for target genetic improvement. In addition, the excellent allele combinations existing in the background will be kept in the process of QTL pyramiding, so target genetic improvement can be realized quickly. Moreover, the IL platform provides a chance to rapidly generate multi-line varieties by mixing the ILs with different biotic stress resistant genes, such as those for rice blast and brown planthopper resistance (Koizumi et al., 2004; Li et al., 2013). In some elite backgrounds, multiple ILs have been constructed by different institutions (Table 1), and global sharing of these resources would accelerate the construction of IL platforms and further facilitate the consortium of rice genetic improvement.

## Author Contributions

YX provided the idea for the review. BZ and XQ collected the data and wrote the manuscript. LM assisted in literature collection. YX, XQ, BZ, LM, and BW revised the manuscript. All authors contributed to the article and approved the submitted version.

## Conflict of Interest

The authors declare that the research was conducted in the absence of any commercial or financial relationships that could be construed as a potential conflict of interest.

## Publisher’s Note

All claims expressed in this article are solely those of the authors and do not necessarily represent those of their affiliated organizations, or those of the publisher, the editors and the reviewers. Any product that may be evaluated in this article, or claim that may be made by its manufacturer, is not guaranteed or endorsed by the publisher.
